# Compensatory proliferation of endogenous chicken primordial germ cells after elimination by busulfan treatment

**DOI:** 10.1186/scrt347

**Published:** 2013-11-05

**Authors:** Hyung Chul Lee, Sung Kyu Kim, Tae Sub Park, Deivendran Rengaraj, Kyung Je Park, Hong Jo Lee, Soo Bong Park, Sung Woo Kim, Seong Bok Choi, Jae Yong Han

**Affiliations:** 1 Department of Agricultural Biotechnology and Research Institute for Agriculture and Life Sciences, Seoul National University, Seoul 151-921, South Korea; 2 Animal Biotechnology Division, National Institute of Animal Science, Rural Development Administration, Suwon 441-706, South Korea; 3 Animal Genetic Resources Station, National Institute of Animal Science, Rural Development Administration, San 4-1 Yongsan-ri Unbong-eup, Namwon 590-832, South Korea

## Abstract

**Introduction:**

Primordial germ cells (PGCs) are the major population of cells in the developing bilateral embryonic gonads. Little is known about the cellular responses of PGCs after treatment with toxic chemicals such as busulfan during embryo development. In this study, we investigated the elimination, restorative ability, and cell cycle status of endogenous chicken PGCs after busulfan treatment.

**Methods:**

Busulfan was emulsified in sesame oil by a dispersion-emulsifying system and injected into the chick blastoderm (embryonic stage X). Subsequently, we conducted flow cytometry analysis to evaluate changes in the PGC population and cell cycle status, and immunohistochemistry to examine the germ cell proliferation.

**Results:**

Results of flow cytometry and immunohistochemistry analyses after busulfan treatment showed that the proportion of male PGCs at embryonic day 9 and female PGCs at embryonic day 7 were increased by approximately 60% when compared with embryonic day 5.5. This result suggests the existence of a compensatory mechanism in PGCs in response to the cytotoxic effects of busulfan. Results of cell cycling analysis showed that the germ cells in the G_0_/G_1_ phase were significantly decreased, while S/G_2_/M-phase germ cells were significantly increased in the treatment group compared with the untreated control group in both 9-day-old male and female embryos. In addition, in the proliferation analysis with 5-ethynyl-2′-deoxyuridine (EdU) incorporation, we found that the proportion of EdU-positive cells among VASA homolog-positive cells in the 9-day embryonic gonads of the busulfan-treated group was significantly higher than in the control group.

**Conclusions:**

We conclude that PGCs enter a restoration pathway by promoting their cell cycle after experiencing a cytotoxic effect.

## Introduction

The continuous maintenance of future generations in living organisms is preserved by germ cell development. Thus, germ cell research is important to advance infertility treatments and perform developmental studies. Elimination of endogenous germ cells has been widely used in germ cell transplantation studies (for clinical purposes) and germline chimera production (for research purposes). Several methods, including gamma ray irradiation, X-ray irradiation [1-3], and busulfan administration [4-6], have been developed to eliminate endogenous germ cells in different vertebrate species. These methods primarily induce DNA damage in target cells, resulting in loss of all cellular mechanisms and ultimately cell destruction. Busulfan is an alkylating agent that can induce target cell apoptosis when administered to cells or tissues. Until recently, busulfan treatment was the preferred method of eliminating germ cells. Although busulfan administration can induce side effects including lethality, sterility and teratogenicity [7], the majority of studies have applied busulfan to eliminate germ cells in mouse and rat testis because of its relatively higher cytotoxicity to target cells. After busulfan administration, testicular germ cells undergo apoptosis; however, small populations of spermatogonial stem cells survive in mice [8]. These surviving spermatogonial stem cells may be involved in restoration of the germ cell population after reduction or withdrawal of busulfan toxicity [9].

Primordial germ cells (PGCs) are the precursors of germ cells in most vertebrates and play an important role in early embryonic germ cells [10]. Elimination of PGCs by busulfan administration can be performed in early chicken embryos because isolation and manipulation of PGCs from these embryos is simple compared with other vertebrate embryos. In chickens, PGCs originate in the epiblast and migrate through the hypoblast and blood to reach embryonic gonads. Busulfan administered to chicken eggs at Eyal-Giladi and Kochav stage X [11] successfully eliminated all endogenous PGCs in the embryos. After busulfan treatment, donor PGCs injected into the embryos migrated and colonized on the recipient gonads. The proportion of donor-derived offspring was also increased significantly [5,12]. However, little is known about the cellular responses of PGCs after busulfan treatment. In the present study, we conducted flow cytometric analysis to evaluate changes in the PGC proportion and cell cycle status after busulfan treatment in chickens.

## Methods

### Experimental animal care

The care and experimental use of chickens were approved by the Institute of Laboratory Animal Resources, Seoul National University (SNU-070823-5). White Leghorn chickens were maintained according to a standard management program at the University Animal Farm, Seoul National University, Korea. The procedures for animal management, reproduction, and embryo manipulation adhered to the standard operating protocols of our laboratory.

### Survival and hatching rates

To measure survival rates, egg candling was performed for each egg during the observation period. Properly developing eggs were identified based on the clear demarcation of light and dark side within the egg and the formation of a network of blood vessels reaching toward the air space. Unfertilized eggs at day 3 were removed from the data and hatching of the eggs occurred at approximately day 21.

### Busulfan emulsification

**Figure 1:**
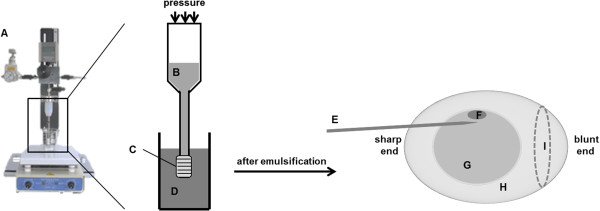
**Schematic diagram of the methods for busulfan emulsification and injection into eggs. (A)** Internal pressure-type micro kit (IMK-20; MCtech, Siheung, Korea). **(B)** Busulfan solubilized in 10% *N*,*N*-dimethylformamide in phosphate-buffered saline. **(C)** Hydrophobic membrane with 10-μm pore diameter. **(D)** Sesame oil with polyglycerol polyricinoleate. **(E)** Sharp needle. **(F)** Blastoderm. **(G)** Egg yolk. **(H)** Egg white. **(I)** Air space.

**Figure 2:**
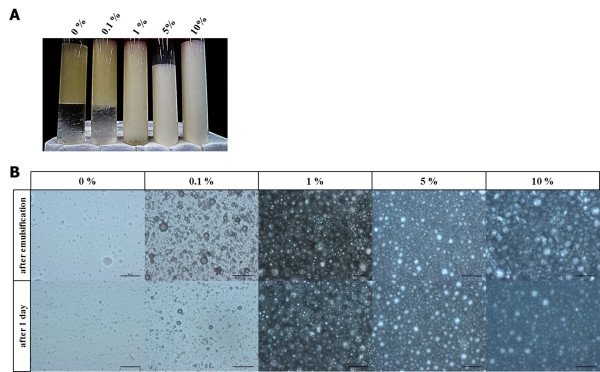
**Increase in particle size uniformity according to polyglycerol polyricinoleate concentration. (A)** Solution feature emulsified with sesame oil containing various polyglycerol polyricinoleate concentrations. **(B)** Particle size uniformity after emulsification and after 1 day (scale bar = 100 μm).

Emulsification of busulfan and injection into chicken embryos was performed as described by Nakamura and colleagues [5], with minor modifications. A schematic diagram of busulfan emulsification and injection into eggs is shown in Figure 1. Approximately 40 mg busulfan (Sigma-Aldrich, St Louis, MO, USA) were dissolved in 1 ml *N*,*N*-dimethyl formamide (Merck, Darmstadt, Germany) and diluted 10-fold in phosphate-buffered saline (PBS). For emulsification, an internal pressure micro kit (IMK-20; MCtech, Siheung, Korea) was used as a dispersion-emulsifying system with a tube-shaped Shirasu porous glass (SPG; pore diameter, 10 μm) membrane. The dispersed phase inside the SPG membrane was filled with busulfan-solubilized solution, and the continuous phase outside the SPG membrane was filled with the same volume of sesame oil (Santa Cruz Biotechnology, Inc., Santa Cruz, CA, USA) with 1% polyglycerol polyricinoleate (PGPR90; Danisco, Denmark) (Figure 2). The internal pressure was injected using nitrogen gas while stirring the continuous phase with a rotator. The final concentration of busulfan in the emulsion was 2 μg/μl in sesame oil containing 1% PGPR90. To optimize the concentration of PGPR90, particle size uniformity and the color of the emulsified solution with different concentrations of PGPR90 were observed at time 0 and 1 day after emulsification. Newly laid White Leghorn eggs at Eyal-Giladi and Kochav stage X were placed horizontally 1 hour before injection. The optimal dose of busulfan was determined based on Nakamura and colleagues [5]. A small hole was made at the sharp end of eggs to avoid air cell damage and 50 μl busulfan emulsion (100 μg busulfan) were injected into the yolk under the blastoderm through a small hole using a sharp needle. After injection, the hole was sealed and the eggs were incubated at 37°C with 50 to 60% relative humidity until the gonads were isolated at embryonic days 5.5, 7, 9 and 15.

### 5-Ethynyl-2′-deoxyuridine incorporation

To examine the proliferation activity of germ cells, approximately 10 μl of 10 mM 5-ethynyl-2′-deoxyuridine (EdU) in PBS was injected into the extra-embryonic blood vessels 4 hours before embryonic day 9. After injection, the eggs were sealed with Parafilm and incubated until the completion of embryonic day 9.

### Immunohistochemistry

After collection of 5.5-day-old and 9-day-old embryos treated with busulfan at stage X, the abdomen of the embryos was carefully dissected under a stereomicroscope and the gonads were collected with sharp tweezers [13]. Whole gonads were then cryosectioned (thickness, 10 μm) or paraffin-sectioned (thickness, 6 μm) and stored for immunostaining. For the immunostaining analysis, gonadal sections (after deparaffinization for paraffin-sectioned tissues) were washed three times with PBS and blocked with blocking buffer, which was composed of PBS containing 5% goat serum and 1% bovine serum albumin, for 1 hour at room temperature. Sections were then incubated at 4°C overnight with rabbit anti-cVASA (chicken VASA homolog) antibody to detect germ cells. After washing three times with PBS, sections were incubated with secondary antibodies labeled with phycoerythrin (PE) or fluorescein isothiocyanate (Santa Cruz Biotechnology) for 4 hours at room temperature. To detect incorporated EdU, sections were further stained for Click-iT detection with Alex Fluor 594 (C10339; Invitrogen, Carlsbad, CA, USA) according to the manufacturer’s instructions. Sections were then mounted with Prolong Gold anti-fade reagent with 4',6-diamidino-2-phenylindole (Invitrogen, Carlsbad, CA, USA) and visualized using fluorescence microscopy.

### Flow cytometry

For flow cytometry, whole gonads from busulfan-treated embryos at day 5.5, day 7, day 9 and day 15 were disassociated by gentle pipetting in 0.05% (v/v) trypsin solution supplemented with 0.53 mM ethylenediamine tetraacetic acid, fixed with 4% paraformaldehyde and permeabilized. Cells were then suspended in PBS containing 1% bovine serum albumin and strained through a cell strainer (40 μm, BD Falcon; Becton Dickinson, Franklin Lakes, NJ, USA). Cell aliquots were incubated in 500 μl of 1% bovine serum albumin in PBS containing primary antibodies (chicken VASA) on ice for 30 minutes. After washing with PBS, cells were incubated in fluorescein isothiocyanate-conjugated secondary antibodies on ice for another 20 minutes. For cell cycle analysis, RNase treatment and propidium iodide staining were performed. Flow cytometry was performed on a FACSAria III (Becton Dickinson). All subsequent analyses were performed using FlowJo software (Tree Star, Ashland, OR, USA) and Modifit LT cell cycle analysis software (Verity Software House, Topsham, ME, USA).

### Statistical analysis

Statistical analysis was performed using Student’s *t* test in the SAS version 9.3 software (SAS Institute, Cary, NC, USA). The significance of differences between control and treatment groups was analyzed using the general linear model (PROC-GLM) in the SAS software. Differences between treatments were considered significant at *P* <0.05.

## Results

### Emulsification conditions for busulfan with PGPR90 by IMK-20

For efficient emulsification of busulfan, PGPR90 was used as an emulsifier. The particle size uniformity was observed under the microscope to confirm the effect of 0.0 to 10.0% PGPR90 on emulsification (Figure 2A). Emulsification did not occur with 0% PGPR90, whereas very low-level emulsification was observed with 0.1% of PGPR90. With 1%, 5% and 10% PGPR90, the particle size uniformity was maintained even after 24 hours (Figure 2B).

### Survival and hatching rate of the chicken embryos after busulfan treatment

To evaluate teratogenic effects of busulfan treatment, we determined the survival and hatching rates during embryonic development. The survival rates of the busulfan treatment group were significantly lower than those of the untreated control group during development. The survival rates of the control and busulfan-treated groups showed no differences at day 3 but were significantly lower in the busulfan-treated group after 7 days of incubation (*P* <0.05). Upon hatching, the survival rates of the two groups were significantly different (*P* <0.01) (Table 1). Mean hatching rates of the untreated control and busulfan treatment groups were 84.47 ± 1.49% (*n* = 3, total events = 71) and 61.85 ± 2.59% (*n* = 3, total events = 144), respectively.

**Table 1:** Survival and hatching rates of chicken embryos after busulfan treatment

**Dose (μg)**	**Number of embryos**	**Survival rates of embryos on incubation day (%)**						**Hatched (%)**
		**Day 3**	**Day 7**	**Day 10**	**Day 14**	**Day 17**	**Day 20**	
Untreated controls (0)	71	94.47 ± 1.08	92.96 ± 1.41	89.93 ± 4.17	88.64 ± 3.99	88.64 ± 3.99	85.91 ± 2.81	84.47 ± 1.49
Busulfan treated (100)	144	87.03 ± 3.16	77.65 ± 3.36	69.75 ± 4.93	68.39 ± 5.31	68.39 ± 5.31	67.65 ± 5.01	61.85 ± 2.59
*P* value		0.0902	0.0136	0.0354	0.0381	0.0381	0.0336	0.0016

### Elimination and restoration of PGCs after busulfan treatment

**Figure 3:**
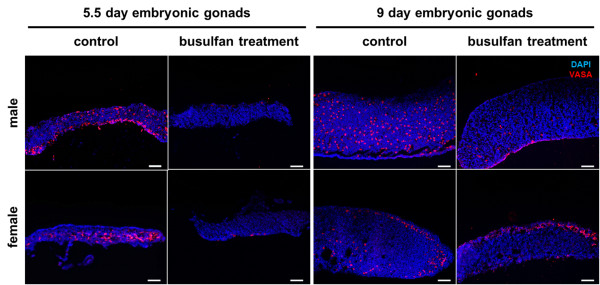
**Elimination and restoration of endogenous primordial germ cells in embryonic gonads by busulfan treatment at stage X.** Immunostaining was performed to detect germ cells in 5.5-day-old and 9-day-old embryonic gonads using an anti-VASA primary antibody and PE-conjugated secondary antibody. Scale bar = 100 μm.

Depletion of PGCs after busulfan treatment was investigated by immunohistochemistry. Whole gonads were collected at embryonic days 5.5 and 9 in both sexes and cryosectioned prior to immunostaining. To identify germ cells, an anti-VASA primary antibody and PE-conjugated secondary antibody were used. At day 5.5, numerous VASA-positive PGCs were dispersed in the gonads of the male and female control group (Figure 3). However, the number of VASA-positive PGCs was greatly decreased in male and female gonads of the busulfan-treated group. At day 9, VASA-positive germ cells were dispersed throughout the male gonads and dispersed in the cortex region of female gonads. In the busulfan-treated group, few VASA-positive germ cells were observed in male and female gonads. Furthermore, the number of VASA-positive germ cells in busulfan-treated female gonads at day 9 was slightly higher than that of busulfan-treated female gonads at day 5.5 (Figure 3).

**Figure 4:**
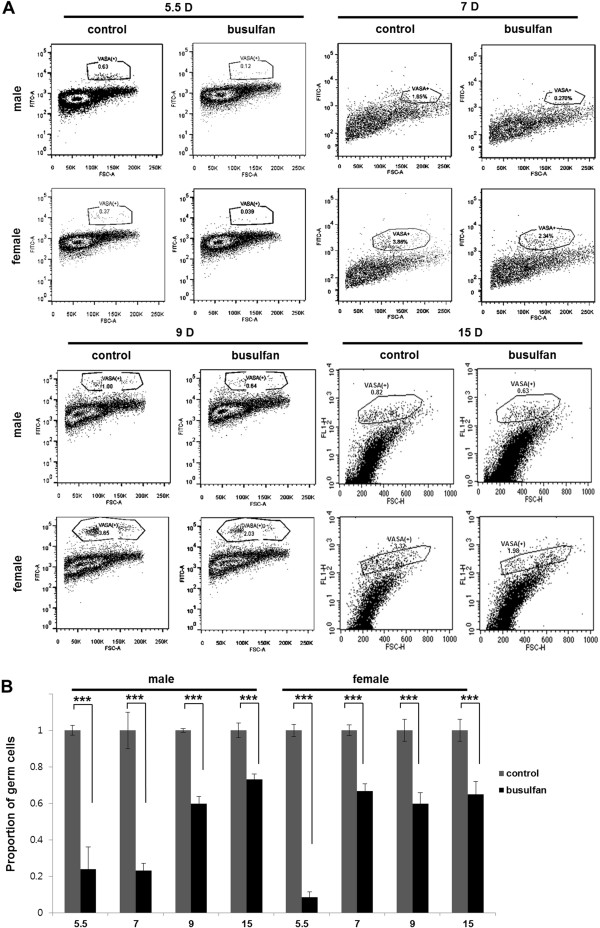
**Proportion of primordial germ cells (PGCs) in the embryonic gonads after busulfan treatment at stage X. (A)** Representative flow cytometry dot plots of day 5.5, day 7, day 9 and day 15 embryonic gonadal cells labeled with anti-VASA. **(B)** Proportion of PGCs in the embryonic gonads of the busulfan-treated group. Data were normalized to the proportion of PGCs in the control. Bars indicate standard error of the mean of triplicate analyses. ***P <0.001, significant difference compared with control.

To examine the proportion of PGCs after busulfan treatment, VASA-positive cells in the embryonic gonads were analyzed by flow cytometry. The mean proportions of PGCs normalized to control PGCs in whole gonads at days 5.5, 7, 9 and 15 are shown in Figure 4. In day 5.5 embryonic gonads, the proportion of PGCs was decreased significantly after busulfan treatment (male, 24%; female, 8%; normalized to control, *n* = 3). In day 7, 9 and 15 embryonic gonads, the proportion of PGCs was also decreased significantly after busulfan treatment (male, 23%, 60% and 71%, respectively; female, 67%, 60% and 65%, respectively, normalized to control, *n* = 3). The rates of VASA-positive PGCs in all busulfan-treated groups regardless of sex or developmental stage were significantly lower than those in the control groups (*P* <0.001) (Figure 4). However, the proportion of PGCs in the busulfan treatment group was significantly increased at embryonic day 9 in male embryos and at embryonic day 7 in female embryos compared with embryonic day 5.5 (Figure 4). Consistent with this germ cell recovery phenomenon, chickens in the busulfan-treated group produced functional sperms or eggs when they reached sexual maturity (*n* = 5 for male and *n* = 3 for female).

### Cell cycle regulation after busulfan treatment

**Figure 5:**
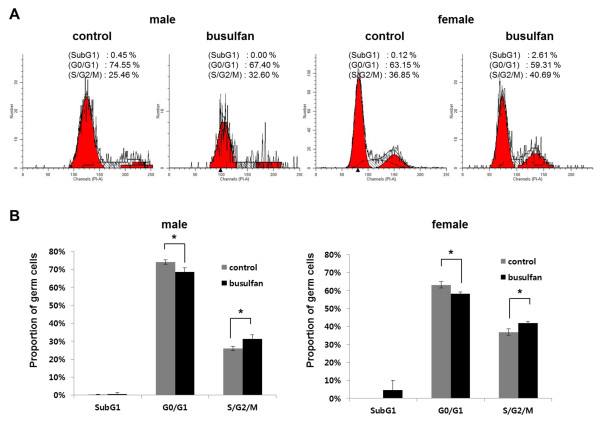
**Cell cycle analysis in primordial germ cells of 9-day-old embryonic gonads after busulfan treatment at stage X.** VASA-positive cell populations in embryonic gonads derived from flow cytometry were evaluated in terms of cell cycle phase. **(A)** Results of representative cell cycle evaluations in males and females. **(B)** Results of replicate cell cycle evaluations in males and females. Bars represent the standard error of the mean of triplicate analyses. *P <0.05, significant difference compared with control.

To examine changes in the cell cycle of PGCs during the recovery period after busulfan treatment, the cell cycle in VASA-positive PGCs of day 9 gonads was evaluated by flow cytometry using propidium iodide staining. Representative and replicate cell cycle results in the PGCs of day 9 gonads after busulfan treatment are shown in Figure 5A and 5B, respectively. In both males and females, the proportion of PGCs in the quiescent phase (G_0_/G_1_) of the busulfan treatment group was significantly decreased compared with the control group (male, 74.03 ± 0.68% to 68.65 ± 1.27%; female, 63.13 ± 1.03% to 58.17 ± 0.61%, *n* = 3). In contrast, the proportion of PGCs in the proliferative phase (S/G_2_/M) of the busulfan-treated group was significantly increased compared with the control group (male, 25.91 ± 0.68% to 31.35 ± 1.27%; female, 36.87 ± 1.03% to 41.83 ± 0.61%, *n* = 3). The proportion of PGCs in the sub-G_1_ phase did not show significant changes between two groups in both males and females.

### Proliferation of restored PGCs after busulfan treatment

**Figure 6:**
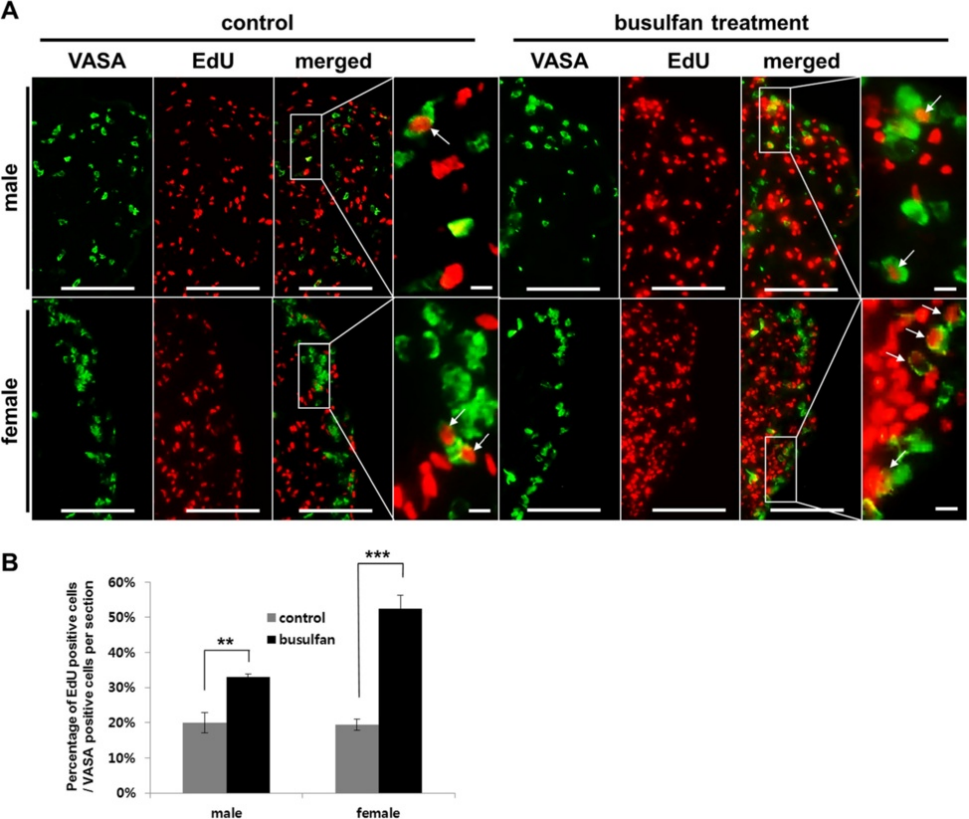
**Proliferation of primordial germ cells (PGCs) in 9-day-old embryonic gonads after busulfan treatment at stage X.** To detect proliferating PGCs, embryonic gonads were isolated at 9 days (4 hours after 5-ethynyl-2′-deoxyuridine (EdU) incorporation) and sectioned for staining with anti-VASA and EdU **(A)**. Scale bars = 100 μm (common views) and 10 μm (magnified views). **(B)** Percentage of EdU-positive cells among VASA-positive cells in the embryonic gonads. Four embryos were evaluated for both sexes. **P <0.01 and ***P <0.001, significant difference compared with control.

To examine proliferation activity of the restored PGCs in the busulfan-treated group, EdU-incorporated 9-day-old embryonic gonads were isolated and immunostained with anti-VASA and EdU. Results showed that EdU-incorporated cell nuclei in the male and female gonads of busulfan-treated groups were increased when compared with control groups (Figure 6A). Furthermore, we investigated the number of proliferating germ cells by counting the number of EdU-positive cells among VASA-positive cells. The number of proliferating germ cells increased by about 15% in busulfan-treated male gonads compared with the control. Similarly, the number of proliferating cells increased by about 30% in busulfan-treated female gonads (Figure 6B).

## Discussion

To eliminate endogenous PGCs in chickens by busulfan treatment, a sustained-release emulsion of busulfan using an SPG pumping connector was used in a previous study [5]. Here, we modified the emulsion methods using an internal pressure micro kit with a tube-shaped SPG membrane and PGPR90. Using this method, we could simplify the preparation of solubilized busulfan and obtain an increased hatching rate (61.85%) in the busulfan treatment group when compared with previous studies that used the same busulfan dose [5].

Busulfan, an alkylating agent, has been used for clinical studies of chronic myelogenous leukemia and bone marrow transplantation [14,15]. Generally, busulfan targets slowly proliferating and nonproliferating cells. The mechanism of action of busulfan has been identified as DNA alkylation leading to DNA–DNA cross-linking [16], which causes cell death and/or cellular senescence through the ERK and p38 pathways. Busulfan also functions as a mitogen-activated protein kinase [17]. Conservation of antimitotic pathway across various cell types remains unclear. Busulfan can specifically target and kill germ cells in embryonic gonads or testes, leading to the depletion of endogenous germ cells. PGCs, which are a precursor of gametes, may therefore be a major target for the germ cell depletion and sterilization. To target PGCs, busulfan should be administered at very early embryonic stages during which PGCs are formed. There are about 30 PGCs in the blastoderm of a fertilized hen egg [18]. Therefore, busulfan has been used to produce PGC-mediated germline chimeras by direct injection into blastoderm of fertilized eggs in chickens [5,6,12]. When injected at Eyal-Giladi and Kochav stage X, busulfan efficiently removed endogenous PGCs [11]. To our knowledge, restoration of endogenous PGCs after busulfan treatment has not been reported to date.

In both sexes, the relative PGC ratios of the busulfan-treated group to the normal embryos at 9 days were markedly higher than that those at day 5.5. Also, sexually mature male and female chickens treated with busulfan at stage X were able to produce functional sperms or eggs. These results indicated that germ cells were recovered from the cytotoxic effects of busulfan during development. We thus hypothesized the existence of a compensation mechanism to recover from busulfan toxicity in PGCs. To confirm the increase in PGCs after busulfan treatment, we conducted flow cytometry to enumerate the increase in PGC number. The number of PGCs in the busulfan treatment group recovered to ~60% that of the control group. This suggested the existence of compensation and/or recovery mechanism in response to cytotoxic damage in PGCs, which is one of the characteristics of stem cells. A strong defensive mechanism against cytotoxic damage has been demonstrated in various stem cells, including spermatogonial stem cells [8] and embryonic stem cells [19]. To determine whether this compensation is caused by changes in the cell cycle, we conducted flow cytometry with propidium iodide staining to discriminate nonproliferating and proliferating PGCs after busulfan treatment. The decrease in the proportion of G0/G_1_-phase PGCs and the increase in that of S/G_2_/M-phase PGCs after busulfan treatment indicated that the cell cycle status of some PGC populations changed from quiescent (G_0_) to proliferative (S/G_2_/M) phases. This change in cell cycle status was further confirmed by the proliferation assay with EdU incorporation. We found that the proportion of EdU-positive cells among VASA-positive cells was significantly higher in the busulfan-treated group.

Our results could be interpreted in two ways: first, a subpopulation of PGCs with stem cell characteristics proliferated, while the majority of PGCs underwent apoptosis after busulfan treatment; or second, proliferation of existing PGCs after busulfan treatment suggested that PGCs possess defensive mechanisms against cytotoxicity. Consistent with the first interpretation, there exists a side population of PGCs in mice [20], which have a greater ability to develop into pluripotent stem cells [21]. In addition, side population cells that differentiated from PGCs were enriched in spermatogonia of developing mice testes [22]. However, the relationship between subpopulations of PGCs and proliferating PGCs after cytotoxic effects was not investigated and little is known about the existence of side population cells in chicken germ cells. Consistent with the second interpretation, conserved expression of several pluripotency-related genes [23,24] and microRNAs [25] were identified in PGCs. The potential of PGCs to transform into pluripotent embryonic germ cells [13,26] indicates that PGCs maintain their undifferentiated state and stem cell attributes in their genetic status. To understand the compensation and/or restoration mechanisms of chicken PGCs, it is necessary to characterize the proliferating PGC subpopulation in busulfan-treated chicken gonads.

## Conclusions

Our data suggest that endogenous PGCs can recover from the cytotoxic effects of busulfan. The cell cycle status of PGCs shifted to a lower proportion in the G_0_/G_1_ phase and a higher proportion in the S/G_2_/M phase after busulfan treatment, which indicates that the recovery of PGCs is strongly associated with the cell cycle transition. Our data increase our understanding of PGCs and provide an important basis for germ cell plantation studies.

## Abbreviations

EdU: 5-ethynyl-2′-deoxyuridine; PBS: phosphate-buffered saline; PGC: primordial germ cells; PGPR90: polyglycerol polyricinoleate; SPG: Shirasu porous glass.

## Competing interests

The authors declare that they have no competing interests.

## Authors’ contributions

HCL participated in manuscript writing, data analysis and interpretation, collection and assembly of data. SKK participated in collection and assembly of data, data analysis and interpretation. S-BP, SWK and S-BC participated in collection and assembly of data. KJP and HJL participated in data analysis and interpretation. TSP and DR participated in manuscript writing. JYH participated in conception and design and final approval of the manuscript. All authors read and approved the final manuscript for publication.
